# Epidemiological and Genomic Landscape of Azole Resistance Mechanisms in *Aspergillus* Fungi

**DOI:** 10.3389/fmicb.2016.01382

**Published:** 2016-09-21

**Authors:** Daisuke Hagiwara, Akira Watanabe, Katsuhiko Kamei, Gustavo H. Goldman

**Affiliations:** ^1^Medical Mycology Research Center, Chiba UniversityChiba, Japan; ^2^Faculdade de Ciências Farmacêuticas de Ribeirão Preto, Universidade de São PauloRibeirão Preto, Brazil

**Keywords:** *Aspergillus fumigatus*, azole resistance, Cyp51A, Cdr1B, tandem repeats, *A. flavus*, *A. niger*, *Aspergillus* section *Fumigati*

## Abstract

Invasive aspergillosis is a life-threatening mycosis caused by the pathogenic fungus *Aspergillus*. The predominant causal species is *Aspergillus fumigatus*, and azole drugs are the treatment of choice. Azole drugs approved for clinical use include itraconazole, voriconazole, posaconazole, and the recently added isavuconazole. However, epidemiological research has indicated that the prevalence of azole-resistant *A. fumigatus* isolates has increased significantly over the last decade. What is worse is that azole-resistant strains are likely to have emerged not only in response to long-term drug treatment but also because of exposure to azole fungicides in the environment. Resistance mechanisms include amino acid substitutions in the target Cyp51A protein, tandem repeat sequence insertions at the *cyp51A* promoter, and overexpression of the ABC transporter Cdr1B. Environmental azole-resistant strains harboring the association of a tandem repeat sequence and punctual mutation of the Cyp51A gene (TR34/L98H and TR46/Y121F/T289A) have become widely disseminated across the world within a short time period. The epidemiological data also suggests that the number of *Aspergillus* spp. other than *A. fumigatus* isolated has risen. Some non-*fumigatus* species intrinsically show low susceptibility to azole drugs, imposing the need for accurate identification, and drug susceptibility testing in most clinical cases. Currently, our knowledge of azole resistance mechanisms in non-*fumigatus Aspergillus* species such as *A. flavus, A. niger, A. tubingensis, A. terreus, A. fischeri, A. lentulus, A. udagawae*, and *A. calidoustus* is limited. In this review, we present recent advances in our understanding of azole resistance mechanisms particularly in *A. fumigatus*. We then provide an overview of the genome sequences of non-*fumigatus* species, focusing on the proteins related to azole resistance mechanisms.

## Introduction

The incidence of fungal infection has increased over the past three decades (Denning, 1998; Dasbach et al., 2000; Kousha et al., 2011; Suzuki et al., 2013; Bitar et al., 2014). This is largely due to the increased number of patients at risk who have received hematopoietic stem cell or solid organ transplantation and immunosuppressive therapy. Although new antifungals have been developed, fungal infections remain a threat to human health. Among filamentous fungal infections, those caused by *Aspergillus* species are the most common. The mortality and morbidity associated with such infections are relatively high, and the predominant causal agent is *Aspergillus fumigatus* (Steinbach et al., 2012). Accordingly, this fungus is regarded as a model pathogen to study many aspects of aspergillosis, such as fungal virulence factors, immune responses, pathology, and drug efficacy. The genome sequence of *A. fumigatus* was determined in 2005 (Nierman et al., 2005), which accelerated our understanding of the molecular mechanisms underlying its pathogenicity (Kwon-Chung and Sugui, 2013; Perez-Nadales et al., 2014; Brown and Goldman, 2016). However, there is still much to be elucidated and the issue of drug resistance has only emerged during the past decade.

The antifungal drugs currently available for the treatment of aspergillosis fall into four categories: pyrimidine, echinocandin, polyene, and azole drugs. Among these, azoles are the first choice drugs in the management and prophylaxis of aspergillosis. Since the first report of an azole-resistant *A. fumigatus* strain in 1997 (Denning et al., 1997), resistant isolates have been detected with increasing incidence worldwide (Chen et al., 2005; Verweij et al., 2007; Howard et al., 2009; van der Linden et al., 2015). It is now widely accepted that azole resistance can develop upon prolonged exposure to azoles at a sub-lethal concentration during the therapy of patients with aspergillosis (particularly with chronic aspergillosis), which is supported by the findings of several studies (Howard et al., 2009; Tashiro et al., 2012; Hagiwara et al., 2014). In addition, environmentally-derived resistance mutations have emerged as a major cause of resistance among strains over the last decade (Snelders et al., 2008; Chowdhary et al., 2013). These mutations involve a combination of tandem repeats (TR) in the *cyp51A* promoter region and amino acid substitution(s) (TR_34_/L98H and TR_46_/Y121F/T289A). The strains harboring such resistance mutations were prevalent among both clinical and environmental settings. As azole resistance is correlated with aspergillosis treatment failure (Howard et al., 2009), a number of studies have focused on the epidemiology, molecular mechanisms, and diagnostic methods relating to this type of resistance. Another emerging issue in the pathology of aspergillosis is that non-*fumigatus* species are being increasingly identified as causal agents of invasive aspergillosis; these include *A. flavus, A. niger, A. terreus*, and *A. calidoustus* as well as other *Aspergillus* species that belong to *Aspergillus* section *Fumigati* (Baddley et al., 2009; Balajee et al., 2009; Krishnan et al., 2009; Tashiro et al., 2011). These species show variable drug susceptibility profiles, imposing the need for clinicians to perform accurate identification and drug susceptibility testing of isolates. Conventional culture methods do not provide an adequate level of specificity and/or sensitivity for accurate diagnosis, making species identification and determination of azole-resistance mutations in the cultured isolates a major challenge. The lack of accurate diagnostic techniques also affects management of patients with aspergillosis caused by azole-resistant *Aspergillus* (Verweij et al., 2015).

Recent advances in DNA sequencing technology have yielded several genomes of *Aspergillus* pathogens and the subsequent detection of sequence variations associated with azole resistance mechanisms. Taken together with epidemiological data, genomic approaches are increasing our understanding of emerging issues in *Aspergillus* pathogenesis. This review aims to summarize the recent findings on azole resistance in *A. fumigatus*, as well as in other related *Aspergillus* pathogens.

## How widely has azole-resistant *Aspergillus* fungi spread?

### Epidemiology of clinically isolated *Aspergillus* fungi

The genus *Aspergillus* comprises 344 species (Samson et al., 2014), and several species have been reported to be pathogenic in humans and animals. It is clear from epidemiological data that *A. fumigatus* is the predominant etiological agent isolated from immune-compromised patients. The second leading cause of aspergillosis is reportedly either *A. flavus* or *A. niger*. Due to the different sources of isolation (country, region, hospital, or ward), and the different clinical manifestations, comparing the frequencies of isolation among different studies is difficult. However, to gain a generalized overview or a trend of the incidence rates, epidemiological data collected from multicenter studies are valuable. Recently, epidemiology data for invasive aspergillosis (IA) was reported, which included a total of 563 patients from 30 intensive care units (ICUs) in eight countries (Taccone et al., 2015). In this study, *A. fumigatus* was the most commonly isolated fungus (*n* = 512, 92%), followed by *A. flavus* (*n* = 19, 3%) and *A. niger* (*n* = 7, 1%). In a 1-year (April 2011 to April 2012) prospective multicenter (18 Belgian hospitals) cohort study, 192 isolates of the *A. fumigatus* complex (87.3%), 13 of the *A. flavus* complex (5.9%), and 10 of the *A. niger* complex (4.5%) were isolated (Vermeulen et al., 2015). In another study of 29 Spanish hospitals (two periods; October 2010 and May 2011), among 278 *Aspergillus* isolates, 156 were identified as *A. fumigatus* (56.1%), 27 were *A. flavus* (9.7%), 26 were *A. terreus* (9.4%), 22 were *A. tubingensis* (7.9%), and 21 were *A. niger* (7.6%; Alastruey-Izquierdo et al., 2013). Prospective cohort studies of transplant-associated fungal infections in the United States carried out by the Transplant-Associated Infection Surveillance Network (TRANSNET) have also revealed that *A. fumigatus* is the leading cause of aspergillosis, followed by *A. flavus, A. niger*, and *A. terreus* (Kontoyiannis et al., 2010; Pappas et al., 2010). Analyzing these data collectively, it is clear that *A. fumigatus* is the predominant causative agent of aspergillosis, but several non-*fumigatus* species were also isolated from patients.

Recently, cryptic species of *Aspergilli* have received much attention owing to advances in the molecular tools for identification. Species that cannot be morphologically distinguished from the leading pathogen of their section are defined as “cryptic” species. *Aspergillus* section *Nigri*, whose members are known as the black aspergilli, is represented by *A. niger*, and includes more than 15 species, including *A. tubingensis* (Abarca et al., 2004). Within the section *Nigri, A. niger*, and *A. tubingensis* are the most common etiological agents of otomycosis, onchomycosis, pulmonary aspergillosis, and aspergilloma (Pappas et al., 2010; Alastruey-Izquierdo et al., 2014; Gheith et al., 2014a; Gautier et al., 2016). *Aspergillus* section *Fumigati*, comprising more than 60 species, also has important clinical implications, with 15 of its species having been reported to be isolated in clinical specimens (Alcazar-Fuoli et al., 2008a; Alastruey-Izquierdo et al., 2014; Lamoth, 2016). Misidentification within the section *Fumigati* has been increasingly reported, with *A. lentulus, A. viridinutans, A. fumigatiaffinis, A. fumisynnematus, A. pseudofischeri, A. hiratsukae*, and *A. udagawae* frequently being reported as *A. fumigatus* (Balajee et al., 2005a,b, 2006; Howard, 2014). Indeed, recent reports demonstrated that the cryptic species including *A. lentulus* and *A. udagawae* accounted for 3–6% of the collection of *Aspergillus* section *Fumigati* isolates (Balajee et al., 2009; Alastruey-Izquierdo et al., 2013; Escribano et al., 2013). These cryptic species in section *Nigri* and section *Fumigati* sometimes show different drug susceptibility profiles and different levels of pathogenicity from those of *A. fumigatus* (Vinh et al., 2009; Coelho et al., 2011; Alastruey-Izquierdo et al., 2014).

Regarding azole resistance, *Aspergillus calidoustus*, belonging to section *Usti*, should be noted, as it shows intrinsic pan-azole resistance. Prior to *A. calidoustus* being identified by specific sequencing as the only pathogenic species in this section, it was often reported as *Aspergillus ustus* (Varga et al., 2008). The incidence of infection caused by *A. calidoustus* was increased in transplant patients under azole prophylaxis (Egli et al., 2012). The TRANSNET study showed that *A. calidoustus* accounted for 2.7% (6/218) of *Aspergillus* species isolates (Balajee et al., 2009), while a population-based survey performed in Spain found 1.4% of the 278 *Aspergillus* species isolates were *A. calidoustus* (Alastruey-Izquierdo et al., 2013). According to these studies, some of these intrinsically azole-resistant *A. calidoustus* strains emerged in the setting of invasive aspergillosis. The growing recognition of azole-resistant cryptic species highlights the clinical need for full and accurate identification and susceptibility testing. In parallel, more focused studies are required to develop a better understanding of these species in the future.

### Prevalence of azole-resistant *Aspergillus* species

The predominant pathogen among *Aspergillus* species, *A. fumigatus*, is intrinsically susceptible to medical azoles. The epidemiological cutoff values (ECVs) for three triazoles, determined by Clinical and Laboratory Standards Institute broth microdilution (CLSI BMD) methods, have been proposed by Pfaller et al. (2009): itraconazole (1 mg/L), voriconazole (1 mg/L), and posaconazole (0.25 mg/L). They showed that 0.2, 0.2, and 0.8% of isolates in a large collection of *Aspergillus* species (*n* = 637) had itraconazole, voriconazole, and posaconazole minimum inhibitory concentrations (MICs) above the ECVs. In a subsequent study (*n* = 1647–2778) by Espinel-Ingroff et al. (2010), the ECVs for *A. fumigatus* were determined by CLSI method as itraconazole (1 mg/L), voriconazole (1 mg/L), and posaconazole (0.5 mg/L). By these criteria, the rates of isolates with itraconazole, voriconazole, and posaconazole MICs outside of the ECVs were 2.6, 3.1, and 2.2%, respectively. The sample size and regional differences in the collections might affect the prevalence of azole-resistant isolates. This study also proposed the ECVs (itraconazole, voriconazole, and posaconazole) for other *Aspergillus* species including *A. flavus* (1, 1, and 0.25 mg/L), *A. terreus* (1, 1, and 0.5 mg/L), *A. niger* (2, 2, and 0.5 mg/L), and *A. nidulans* (1, 2, and 1 mg/L; Espinel-Ingroff et al., 2010). These ECV data could help to characterize *Aspergillus* isolates and to monitor the emergence of azole-resistant strains by *in vitro* antifungal susceptibility testing with CLSI BMD method.

Over the past decade, ongoing azole resistance surveillance reports have been published by several research groups from different countries. A summary of recent reports on the surveillance of major *Aspergillus* species is shown in Table 1. The prevalence of azole-resistant *A. fumigatus* and *A. terreus* strains appears to be largely consistent between the studies. Based on the ECVs determined in the study by Espinel-Ingroff et al. (2010), the prevalence rates for azole-resistant isolates determined using the CLSI method in these studies are listed in Table 2. In these studies, 0.2–2.6, 0.8–3.1, and 0.2–2.2% of *A. fumigatus* isolates were resistant to itraconazole, voriconazole, and posaconazole, respectively, while 0, 0–3.0, and 0–0.3% of *A. terreus* isolates were resistant to itraconazole, voriconazole, and posaconazole, respectively (Table 2). It is notable that *A. flavus, A. niger*, and *A. tubingensis* showed variations in the prevalence of azole resistance between the studies. In particular, high rates of resistance to itraconazole were demonstrated amongst *A. niger* strains, suggesting intrinsic resistance to itraconazole in this species. However, this remains controversial as the effects of regional and individual laboratory conditional differences between the studies cannot be ruled out. Thus, further studies are necessary to draw definite conclusions.

**Table 1:** **Summary of recent reports on azole-resistant ***Aspergillus*** species isolates**.

**References**	**Method**	**Species**	***n***	**ITCZ**		**VRCZ**		**PSCZ**	
				**MIC90**	**Range**	**MIC90**	**Range**	**MIC90**	**Range**
Pfaller et al., 2008	CLSI M38-A2	*A. fumigatus*	553	1	0.12–2	0.5	0.06–4	0.5	0.03–2
		*A. flavus*	76	1	0.12–2	1	0.06–1	0.5	0.06–2
		*A. niger*	59	>8	0.5–>8	1	0.12–2	1	0.12–2
		*A. terreus*	35	0.5	0.12–1	0.5	0.06–1	0.25	0.06–0.5
Baddley et al., 2009	CLSI M38-A2	*A. fumigatus*	181	0.5	0.125–4	0.5	0.125–8	0.125	0.03–1
		*A. niger*	28	1	0.25–1	1	0.5–1	0.25	0.06–0.5
		*A. flavus*	27	0.25	0.06–0.25	0.5	0.125–1	0.125	0.06–0.125
		*A. terreus*	22	0.25	0.03–0.25	0.5	0.25–0.5	0.06	0.03–0.06
Espinel-Ingroff et al., 2010	CLSI M38-A2	*A. fumigatus*	1684–2815	1	0.03–16	1	0.03–16	0.25	<–0.01–4
		*A. flavus*	323–592	0.5	0.03–16	1	0.06–16	0.25	0.03–16
		*A. niger*	366–520	2	0.03–16	2	0.03–32	0.5	0.03–2
		*A. terreus*	330–462	1	0.03–1	1	0.03–32	0.5	0.03–2
		*A. nidulans*	131–143	1	0.03–8	1	0.03–8	1	0.03–8
Shivaprakash et al., 2011	CLSI M38-A2	A. flavus^*1^	188	0.25	0.062–0.5	2	0.5–4	0.25	0.062–0.25
Al-Wathiqi et al., 2013	E-test	*A. flavus*	92			0.25	0.064–0.25	0.25	0.016–0.38
Alastruey-Izquierdo et al., 2013	EUCAST	*A. fumigatus*	156	0.25	0.12–1	1	0.12–2	1	0.25–1
		*A. flavus*	27	1	0.06–1	1	0.12–4	2	0.25–4
		*A. terreus*	26	0.25	0.06–0.25	2	0.5–2	1	0.25–2
		*A. tubingensis*	22	1	0.03–32	2	0.25–2	2	0.25–2
		*A. niger*	21	0.5	0.06–1	1	0.25–2	2	0.25–2
Gheith et al., 2014b	E-test	*A. flavus*	18	0.83	0.25–1	0.25	0.06–0.5	0.25	0.06–0.25
		*A. niger*	17	2	0.38–2	0.13	0.05–0.12	0.25	0.05–0.25
		*A. tubingensis*	9	4.8	0.25–8	0.38	0.064–0.38	0.25	0.047–0.25
Lalitha et al., 2014	CLSI M38-A2	*A. flavus*	32			2	0.25–8		
		*A. fumigatus*	10			1.3	0.25–4		
van Ingen et al., 2015	EUCAST	*A. fumigatus*	952	>16	0.063–>16	>16	0.25–>16	1	0.031–>16
Gautier et al., 2016	E-test	*A. niger*	36	12	0.25–24	0.5	0.064–1	0.5	0.047–1
		*A. tubingensis*	36	32	0.38–32	0.75	0.125–1	0.5	0.047–0.75
Badali et al., 2016	CLSI M38-A2	*A. niger* (clinical)	39	>16	0.25–>16	>16	0.125–>16	0.125	0.016–0.125
		*A. niger* (environmental)	33	>16	0.125–>16	>16	0.125–>16	0.125	0.016–0.25
		*A. tubingensis* (clinical)	20	1	0.25–1	1	0.063–1	0.125	0.016–0.125
		*A. tubingensis* (environmental)	29	>16	0.125–>16	1	0.125-2	0.125	0.016–0.125
Khodavaisy et al., 2016	CLSI M38-A2	*A. flavus* (clinical)	171	0.5	0.031–2	0.5	0.031–8	0.25	0.008–0.5
		*A. flavus* (environmental)	28	1	0.25–1	1	0.25–4	0.125	0.047–0.5
Kachuei et al., 2016	CLSI M38-A2	*A. flavus*	38	0.5	0.063–2	1	0.031–1	0.125	0.008–0.25
Castanheira et al., 2016	CLSI M38-A2	*A. fumigatus*	142	1	0.25–4	0.5	0.12–2	0.5	0.06–1

*1 *Contains clinical and environmental isolates*.

*ITCZ, itraconazole; VRCZ, voriconazole; PSCZ, posaconazole*.

**Table 2:** **Summary of the rates of azole resistance amongst ***Aspergillus*** species**.

**Species**	***n***	**Country**	**Method**	>ECV (%)^*1^			**References**
				**ITCZ**	**VRCZ**	**PSCZ**	
*A. fumigatus*	637	Worldwide	CLSI M38-A2	1/637 (0.2)	5/637 (0.8)	1/637 (0.2)	Pfaller et al., 2009
	1684–2815	Worldwide	CLSI M38-A2	68/2554 (2.6)	88/2778 (3.1)	37/1647 (2.2)	Espinel-Ingroff et al., 2010
*A. flavus*	76	Worldwide	CLSI M38-A2	1/76 (1.3)	0/76 (0)	57/76 (25)	Pfaller et al., 2008
	27	The United States	CLSI M38-A2	0/27 (0)	0/27 (0)	0/27 (0)	Baddley et al., 2009
	323–592	Worldwide	CLSI M38-A2	4/536 (0.7)	12/590 (2.0)	18/321 (5.6)	Espinel-Ingroff et al., 2010
	188	India and The Netherlands	CLSI M38-A2	0/188 (0)	49/188 (26.1)	0/188 (0)	Shivaprakash et al., 2011
	171	Iran (clinical)	CLSI M38-A2	1/171 (0.6)	1/171 (0.6)	4/171 (2.4)	Khodavaisy et al., 2016
	28	Iran (environmental)	CLSI M38-A2	1/28 (3.6)	1/28 (3.6)	1/28 (3.6)	Khodavaisy et al., 2016
*A. niger*	59	Worldwide	CLSI M38-A2	14/59 (23.7)	0/59 (0)	9/59 (15.3)	Pfaller et al., 2008
	28	The United States	CLSI M38-A2	0/28 (0)	0/28 (0)	0/28 (0)	Baddley et al., 2009
	366–520	Worldwide	CLSI M38-A2	41/427 (8.8)	5/479 (1.0)	19/325 (5.2)	Espinel-Ingroff et al., 2010
	39	Iran (clinical)	CLSI M38-A2	7/39 (17.9)	6/39 (15.4)	0/39 (0)	Badali et al., 2016
	33	Iran (environmental)	CLSI M38-A2	14/33 (42.4)	11/33 (33.3)	0/33 (0)	Badali et al., 2016
*A. terreus*	35	Worldwide	CLSI M38-A2	0/35 (0)	0/35 (0)	0/35 (0)	Pfaller et al., 2008
	22	The United States	CLSI M38-A2	0/22 (0)	0/22 (0)	0/22 (0)	Baddley et al., 2009
	330–462	Worldwide	CLSI M38-A2	0/369 (0)	14/462 (3.0)	1/330 (0.3)	Espinel-Ingroff et al., 2010
A. tubingensis^*2^	20	Iran (clinical)	CLSI M38-A2	0/20 (0)	0/20 (0)	0/20 (0)	Badali et al., 2016
	29	Iran (environmental)	CLSI M38-A2	11/29 (37.9)	0/29 (0)	0/29 (0)	Badali et al., 2016
*A. nidulans*	131–143	Worldwide	CLSI M38-A2	9/141 (6.3)	2/139 (1.4)	3/129 (2.3)	Espinel-Ingroff et al., 2010

*ECV, epidemiological cutoff values; ITCZ, itraconazole; VRCZ, voriconazole; PSCZ, posaconazole*.

*1 *: ECVs proposed by Espinel-Ingroff et al. (2010) were used for this analysis (see text)*.

*2 *: ECVs for A. niger were applied as ECVs have not been established for A. tubingensis*.

In addition to the non-*fumigatus* species, the MICs for azoles were determined in some species of *Aspergillus* section *Fumigati* (Table 3). Isolates of *A. lentulus, A. udagawae*, and *A. viridinutans* with high MICs (>1 mg/L) for itraconazole and/or voriconazole were detected in most of these studies. Although these data were suggestive of intrinsic resistance to the azoles in these species, it should not be overlooked that the susceptibilities were variable among isolates. It is particularly noteworthy that posaconazole appears to retain remarkable antifungal activity against these cryptic species, and that novel azole drug isavuconazole also shows significant activity in these species (Datta et al., 2013).

**Table 3:** **Summary of the MICs for ***Aspergillus*** section Fumigati**.

**References**	**Species**	***n***	**ITCZ**		**VRCZ**		**PSCZ**	
			**MIC90**	**Range**	**MIC90**	**Range**	**MIC90**	**Range**
Tamiya et al., 2015	*A. fumigatus*	69	0.5	–	1	–	–	–
	*A. lentulus*	8	2	–	8	–	–	–
	*A. udagawae*	9	8	–	8	–	–	–
Datta et al., 2013	*A. lentulus*	15	2	0.5–2	2	0.5–2	–	–
	*N. udagawae*	10	1	0.25–1	1	0.25–1	–	–
Escribano et al., 2013	*A. lentulus*	6	–	1–2	–	1–4	–	0.5–1
	*N. udagawae*	2	–	1	–	1	–	0.5
	*A. viridinutans*	1	–	4	–	4	–	0.5
Vinh et al., 2009	*N. udagawae*	4	–	1–4	–	1–116	–	0.25–0.5
Gürcan et al., 2013	*A. lentulus*	1	–	–	–	0.25	–	0.125
Lago et al., 2014	(A. lentulus)^*1^	7	–	1	–	2–4	–	0.125–0.25
	(A. fumigatus)^*1^	8	–	0.25–1	–	0.25–0.5	–	0.031–0.25
Alastruey-Izquierdo et al., 2014	*A. lentulus*	26	16	0.12–16	8	0.25–16	0.5	0.03–1
	*A. udagawae*	5	1	0.25–1	4	2–4	0.25	0.12–0.25
Mortensen et al., 2011	*A. fumigatus*	107	1	<–0.03–>4	0.5	0.125–2	0.25	<–0.03–>4
	*A. lentulus*	1	–	2	–	2	–	0.5
Balajee et al., 2009	*A. lentulus*	4	–	0.25–0.5	–	1–4	–	0.25
	*A. udagawae*	3	–	0.25–2	–	0.25–2	–	0.125–0.25
Alcazar-Fuoli et al., 2008a	*A. lentulus*	14	–	(0.43–16)^*2^	–	(3–7.5)^*2^	–	(0.12–2)^*2^
	*A. viridinutans*	2	–	(14.4–16)^*2^	–	(4)^*2^	–	(0.25–0.41)^*2^

*1 *: isolated from the environment*.

*2 *: Geometric means (GMs) were used*.

*ITCZ, itraconazole; VRCZ, voriconazole; PSCZ, posaconazole*.

Many surveys focused particularly on *A. fumigatus* have been conducted, as azole resistance mechanisms have been intensively investigated in this fungus. An overview of the prevalence of azole-resistant strains was presented in two excellent reviews both published in 2013 (Lelièvre et al., 2013; Vermeulen et al., 2013). The literature cited in the review by Vermeulen et al. (2013) revealed that the overall azole resistance rate of *A. fumigatus* ranged from 0.6 to 27.8% across the studies. Again the prevalence rates varied between studies (countries), likely due to differences in region and disease manifestation in patients. The emergence and spread of “environmental resistance mechanisms” during the past decade might also have affected the prevalence data. Accordingly, a research group from the Netherlands most recently published a survey of azole-resistant *A. fumigatus*, in which 364 of 952 clinical strains (38.2%) isolated by, or referred to, the laboratory from 2010 to 2013 were resistant to azoles (van Ingen et al., 2015). Among them, 225 (23.6%) and 98 (10.3%) strains possessed the environmental resistance mechanisms TR34/L98H and TR46/Y121F/T289A, respectively. Another group from the Netherlands reported that 21 azole-resistant strains (20.0%) out of 105 isolates included 13 strains harboring the TR34/L98H allele (12.4%) and three strains harboring the TR46/Y121F/T289A allele (2.9%; Fuhren et al., 2015). The isolation rate of environmentally-derived azole-resistant *A. fumigatus* strains seems to be constantly increasing, threatening the effectiveness of current frontline antifungal therapy against aspergillosis.

A multicenter epidemiological study carried out by the Surveillance Collaboration on Aspergillus Resistance in Europe (SCARE) network provided a more general understanding of the prevalence of azole-resistant *Aspergillus* strains (van der Linden et al., 2015). In total, 22 centers from 19 countries participated the study, and 3788 *Aspergillus* isolates were collected between January 2009 and January 2011. Of these, 2941 isolates (77.6%) belonged to the *A. fumigatus* complex, 60 of which showed azole resistance. Forty-seven of these azole-resistant isolates were *A. fumigatus sensu stricto*, and strains with environmental resistance mechanisms TR34/L98H or TR46/Y121F/T289A accounted for 55.3% (*n* = 26) of isolates. These strains were recovered from six countries: Austria, Belgium, Denmark, France, Italy, and The Netherlands. Recently, strains containing the TR34/L98H and TR46/Y121F/T289A mutations have also been identified in the United States, Columbia, Taiwan, and Japan (Wu et al., 2015; Hagiwara et al., 2016; Le Pape et al., 2016; Wiederhold et al., 2016). These reports highlight a global risk for resistant *A. fumigatus* strains.

## Azole resistance mechanisms in *A. fumigatus*

**Figure 1:**
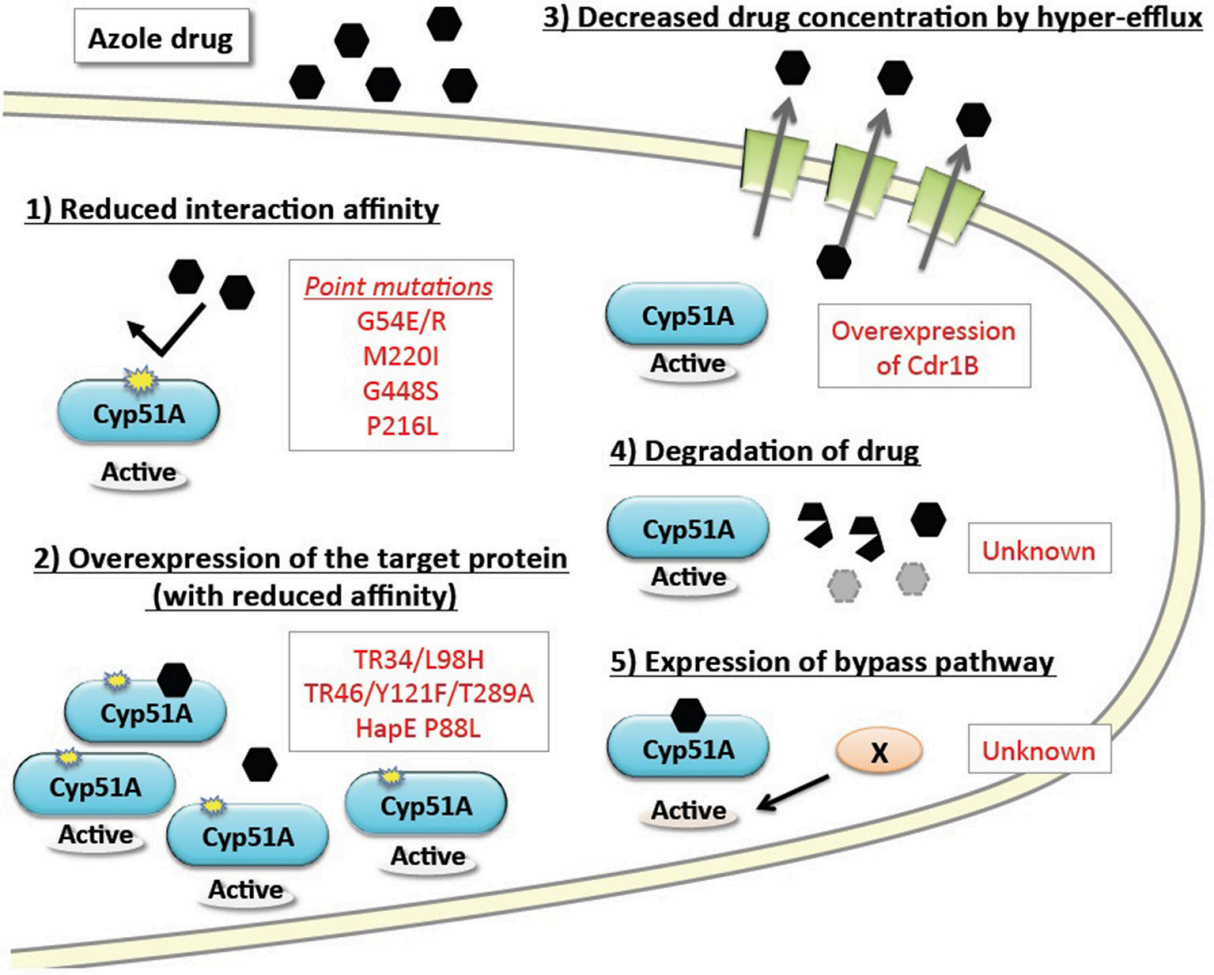
**Basic drug resistance mechanisms and the corresponding azole resistance mechanisms in ***A. fumigatus*****. Drug resistance mechanisms are categorized into five basic types. The underlying molecular mechanisms identified in *A. fumigatus* are shown in red text.

The basic resistance mechanisms of microbial cells to growth-inhibiting drugs are depicted in Figure 1. The mechanisms are categorized as: (1) reduced interaction affinity of the target protein to the drugs, (2) overexpression of the target protein in the cells, (3) decreased drug concentration by boosted efflux system, (4) intra- or extra-cellular degradation of the drugs, and (5) alternative pathways bypassing the drug effects. The following section summarizes recent advances in our understanding of each of these resistance mechanisms, particularly in regard to *A. fumigatus*. Furthermore, we review several attempts to reproduce the resistance mutations under laboratory conditions, and new approaches to identifying the responsible mutations by genome comparison.

### CYP51 proteins

**Figure 2:**
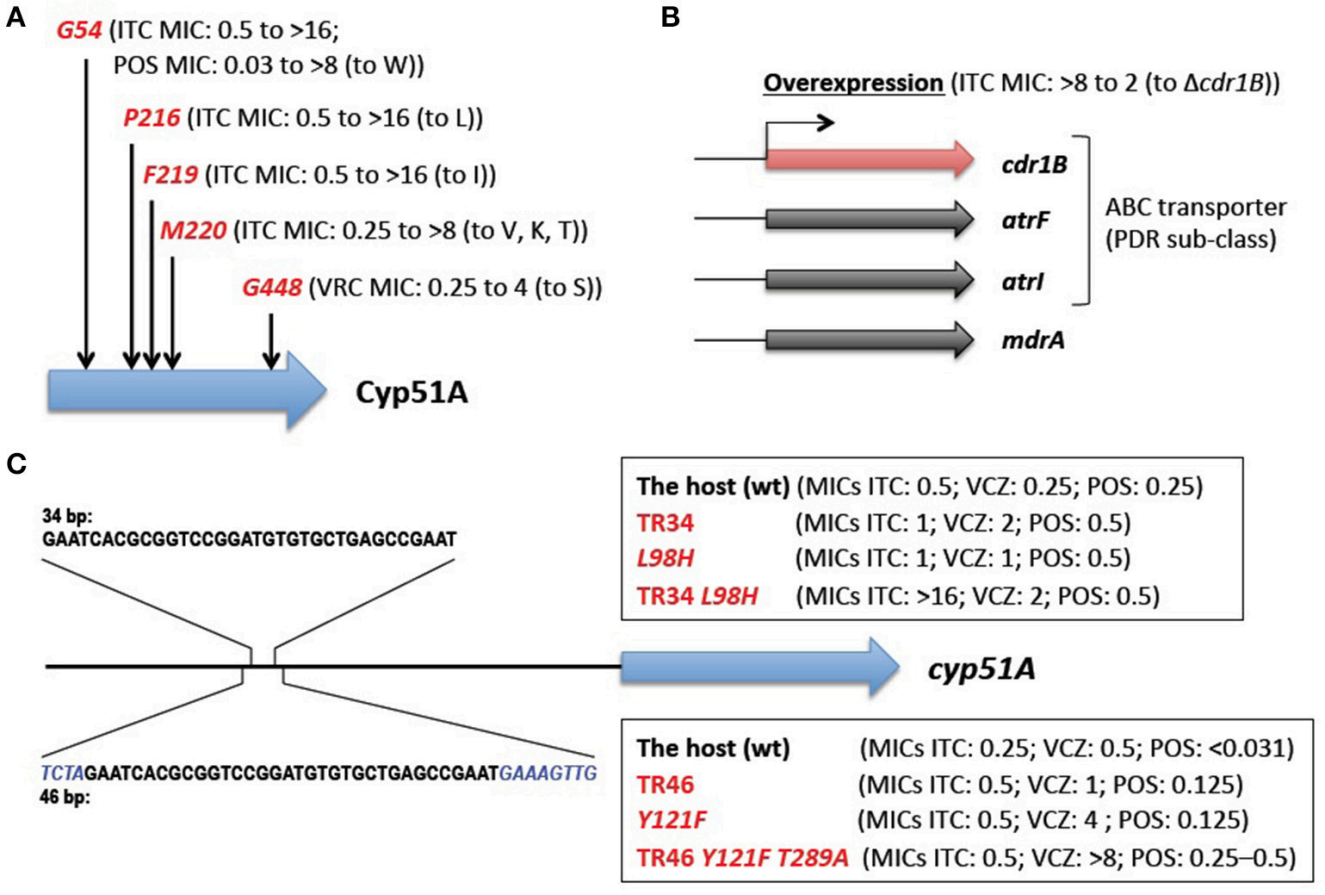
**Known azole resistance mechanisms in ***A. fumigatus*****. **(A)** Amino acid substitutions responsible for azole resistance. G54, P216, F219, M220, and G448 show the position at which amino acid changes resulted in azole resistance. The MICs of the strains harboring the change (to the indicated amino acid) are shown in parentheses (Mann et al., 2003; Mellado et al., 2004; Camps S. M. et al., 2012; Krishnan-Natesan et al., 2012). **(B)** The efflux transporters related to azole resistance. The MIC of the strain with overexpression of the *cdr1B* gene is shown in parentheses (Fraczek et al., 2013). **(C)** The tandem repeat sequences in a *cyp51A* promoter. The none-overlapping bases are shown in italics. The MICs of the strains harboring TR34 or TR46 and/or amino acid change(s) are shown in parentheses (Snelders et al., 2011, 2015). ITCZ, itraconazole; PSCZ, posaconazole; VRCZ, voriconazole.

The main mechanisms of azole resistance were elucidated based on the identification of numerous mutations in resistant *A. fumigatus* isolates. The most frequent mutations detected were related to the target protein Cyp51A: 14α-demethylase. This enzyme is involved in ergosterol biosynthesis and sterol metabolism, and plays an important role in *Aspergillus*. *A. fumigatus* has a paralogous protein Cyp51B. Both proteins are capable of complementing the lethality of a *Saccharomyces cerevisiae erg11/cyp51* (sterol 14α-demethylase) mutant (Martel et al., 2010), indicating the functionality of the Cyp51 proteins. Neither Cyp51A nor Cyp51B is individually essential for *A. fumigatus* growth (Garcia-Effron et al., 2005; Mellado et al., 2005), but the attempted inactivation of both genes was lethal (Hu et al., 2007). Azoles interact with and inhibit the Cyp51 proteins (Warrilow et al., 2015), which in turn reduce the ergosterol content and impair sterol metabolism in the cells (Alcazar-Fuoli et al., 2008b), resulting in a fungistatic (under low azole concentrations), or a fungicidal (under high azole concentrations) effect. Single amino acid substitutions at G54, P216, F219, M220, and G448 in the Cyp51A protein have been well-described to confer azole resistance (Mann et al., 2003; Mellado et al., 2004; Camps S. M. et al., 2012; Krishnan-Natesan et al., 2012; Figure 2A). Besides these “hotspots,” amino acid changes at other positions (Y121, G138, and Y431) were also found in the Cyp51A protein of resistant strains (Albarrag et al., 2011; Lescar et al., 2014). These resistance mutations tend to arise during prolonged treatment of chronic aspergillosis with azole drugs. This was well-exemplified by several cases, in which azole-susceptible and azole-resistant isolates were serially isolated from one individual patient (Howard et al., 2009; Hagiwara et al., 2014). It is worth noting that such resistance mutations have never been reported to date in the Cyp51B protein. Instead, high-level induced expression and constitutive overexpression of the *cyp51B* gene were observed in azole-resistant clinical isolates (Buied et al., 2013). Although these findings await further genetic verification, they raise the possibility of the involvement of Cyp51B in azole resistance as a non-Cyp51A-mediated resistance mechanism in clinical settings.

### Efflux transporters

The overexpression of efflux transporters has been well-documented in yeasts. *A. fumigatus* contains at least 49 genes encoding the ATP-binding cassette (ABC) transporter (Lelièvre et al., 2013), among which 12 genes show high homology (>30% identities and >50% positive results for >80% of the query protein sequences) with the *S. cerevisiae* PDR5 and PDR15 proteins that are involved in azole resistance (Paul and Moye-Rowley, 2014). These 12 transporters are included in a PDR (also called an ABC-G) sub-class of ABC transporters. Among them, Cdr1B was identified to be overexpressed in azole-resistant strains, and deleting the *cdr1B* gene in one such strain resulted in increased susceptibility to itraconazole (Fraczek et al., 2013; Figure 2B). The other strains with the *cdr1B* gene deleted showed azole hyper-sensitivity (Paul et al., 2013), which indicated that Cdr1B is responsible for azole resistance in *A. fumigatus*. Recently, Dr. Sanglard and his colleagues demonstrated that deletion mutants of two distinct ABC transporters (AtrF, AtrI) and a major facilitator superfamily transporter (MdrA) also showed sensitivity to azoles (Meneau et al., 2016). The expression levels of the *cdr1B* gene (also called *abcB*) were slightly induced upon voriconazole treatment (Paul et al., 2013), whereas some of the other transporter genes (*abcB*/Afu1g10390, *abcE, mfsA, mfsB*, and *mfsC*) were shown to be upregulated in response to voriconazole (da Silva Ferreira et al., 2006). It therefore appears that other efflux transporters may potentially be involved in clinical azole resistance.

### Environmentally-derived resistance mechanisms

During this decade, *A. fumigatus* azole-resistant strains with a combination of a TR in the promoter region of *cyp51A* and amino acid mutation(s) (TR34/L98H and TR46/Y121F/T289A) have been increasingly reported. These strains have been isolated from patients regardless of their azole treatment history, as well as from the environment. The strains harboring such mutations were isolated in many countries from several continents (Mellado et al., 2007; Snelders et al., 2008; Vermeulen et al., 2012; Chowdhary et al., 2014; Wu et al., 2015; Hagiwara et al., 2016; Wiederhold et al., 2016). The high incidence of resistant strains reported in the Netherlands is particularly alarming (Fuhren et al., 2015; van Ingen et al., 2015). It is now widely accepted that such resistance mechanisms were derived from exposure to azole fungicides in the environment (Snelders et al., 2008).

The molecular mechanisms underlying environmentally-derived mutations were intensively studied by Dr. Melchers's group (Figure 2C). *A. fumigatus* recombinants with different *cyp51A* amino acid substitutions and/or a promoter insertion were constructed (Snelders et al., 2011, 2015). The recombinants harboring TR34 or TR46 showed increased expression of the *cyp51A* gene compared with the parental strains, however, the TR by itself only had a moderate effect on azole susceptibility (itraconazole (ITCZ) MIC: 0.25–0.5 to 0.5–1 mg/L; voriconazole (VRCZ) MIC: 0.25–0.5 to 1–2 mg/L). Site-directed mutagenesis of L98H or Y121F alone also showed a moderate increase in the MICs (ITCZ MIC: 0.25–0.5 to 0.5–1 mg/L; VRCZ MIC: 0.25–0.5 to 1–4 mg/L). Azole resistance levels comparable to those of the clinical isolates (TR34/L98H or TR46/Y121F/T289A) were achieved by a combination of the TR and an amino acid substitution; the recombinant with TR34/L98H showed MICs >16 mg/L for ITCZ and 2 mg/L for VRCZ, and the recombinant with TR46/Y121F showed MICs >8 mg/L for ITCZ and >8 mg/L for VRCZ. Interestingly, the results suggested that T289A was dispensable for full resistance to azoles. Paul et al. (2012) also evaluated the effect of the TR34 element on *cyp51A* gene expression using a promoter-luciferase reporter system. The lack of the 34-bp sequence led to a 90% reduction in the expression level compared with the wild-type promoter, indicating that the 34-bp element played a critical role in maintaining wild-type expression of the *cyp51A* gene. Although repeats of the 34-bp element resulted in a modest increase in expression, the sequence may function as an enhancer element for *cyp51A* gene expression.

Several reports of similar Cyp51 overexpression were also found in plant fungal pathogens (Becher and Wirsel, 2012). The high expression was correlated with the presence of insertions in the promoter region of the *cyp51* gene. A five-time TR of a unique 126-bp sequence was found in the *cyp51A* promoter region in *Penicillium digitatum* (Hamamoto et al., 2000). Furthermore, 553-, 120-, and 65-bp insertions were found in the *cyp51* promoter region in *Venturia inaequalis, Mycosphaerella graminicola*, and *Monilinia fructicola*, respectively (Schnabel and Jones, 2000; Luo and Schnabel, 2008; Cools et al., 2012). Changes in the promoter region of the *cyp51* gene by the presence of insertions or TRs may frequently occur under certain conditions such as long-term exposure to azole fungicides in environmental niches.

### Lessons from *in vitro* evolution and perspectives

To broaden our knowledge of azole resistance mutations, attempts to evolve drug-resistant *A. fumigatus* strains *in vitro* by transferring strains onto plates containing sub-lethal concentration of azoles have been undertaken by several groups. da Silva Ferreira et al. (2004) generated 10 itraconazole-resistant strains (nine showed >16 mg/L of ITCZ MIC and one showed 1 mg/L) by *in vitro* evolution procedure, three of these possessed a G54R point mutation in the Cyp51A protein, and two possessed a M220I point mutation in this protein. Novel mutations (positions N22, T440, and Y491) were also found in the Cyp51A protein of itraconazole-resistant strains, among which the N22D substitution was shown to confer itraconazole resistance to wild-type *A. fumgiatus* by genetic transformation. They also demonstrated that these resistant mutants tended to show increased expression levels of efflux transporter genes. Upon voriconazole exposure in another study, six resistant strains were obtained from three different parental strains under laboratory conditions (Krishnan-Natesan et al., 2012). All of these strains harbored a G448S mutation in the Cyp51A protein and showed MICs of 2–8 mg/L for VRCZ, whereas the ITCZ MIC ranged from 0.25 to 2 mg/L and the PSCZ MIC from 0.0625 to 0.15 mg/L. This suggested that G448S is the predominant mutation conferring resistance to voriconazole. Snelders et al. (2012) exposed distinct *A. fumigatus* strains (wild-type, the strain with TR34, and the strain with L98H) to itraconazole, azole fungicides (bromuconazole, difenoconazole, epoxiconazole, propiconazole, or tebuconazole), or a mixture of the fungicides. In the case of itraconazole exposure, G138C and P216L were identified in the wild-type strain that had evolved by more than three transfers. However, none of the strains gained “environmentally-derived mutations” such as TR34/L98H and TR46/Y121F/T289A. The authors hypothesized that the development of TR34/L98H and TR46/Y121F/T289A mutations might be extremely infrequent in the environment. Recent microsatellite genotyping studies showed that less genetic variation was found among strains harboring TR34/L98H or TR46/Y121F/T289A mutations compared with the set of wild-type isolates (Snelders et al., 2009; Hagiwara et al., 2016). From these studies, it is tempting to speculate that the TR34/L98H and TR46/Y121F/T289A-containing isolates did not arise in distinct strains in various regions, but might have originated from common ancestors. If this is the case, the rapid spread of these resistant strains across the globe in a short period of time is quite surprising and worrisome. At this moment, however, the possibility that there is a preferential genetic background in which “environmental mutations” occur more frequently cannot be ruled out. More extensive studies, potentially including population genetic analyses, might provide insight into this important issue.

### Exploring resistance mutations by whole-genome comparisons

As stated earlier, azole resistance mutations tend to occur during long-term azole treatment (Tashiro et al., 2012). In some cases, multiple strains are serially isolated from one patient at different time points. Resistant isolates theoretically possess the mutation attributed to resistance, which does not exist in the corresponding sensitive isolates. Inspection of the *cyp51A* gene sequence between these isolates has uncovered several mutations in the Cyp51A protein. Furthermore, to identify novel non-*cyp51A* resistance mutations, whole-genome comparisons were conducted between azole-sensitive and azole-resistant cognate isolates. Camps S. M. T. et al. (2012) identified a new mutation in the *hapE* gene by whole-genome comparison and verified its involvement by sexual crossing. HapE encodes a subunit of the CCAAT-binding transcription factor complex, which plays a regulatory role in a wide array of fungal phenotypes. The *cyp51A* expression level in strains with the *hapE* mutation was higher than in the corresponding wild-type strain, which suggested that HapE plays a role in regulating *cyp51A* gene expression. As a presumed CCAAT-box is present in the promoter region of *cyp51A*, further analysis may uncover the mechanism of regulation of the *cyp51A* gene. In addition to this novel resistance mutation, they also detected at least 22 mutations in a pair of strains that were recovered 17 weeks apart. Among them, five of these mutations appeared to be in non-coding regions and 11 were synonymous mutations. This indicated that dynamic alterations, likely irrespective of azole resistance, occur in the *A. fumigatus* genome within its host during infection and treatment. In another study, a large genomic deletion in a region containing 11 genes was identified by whole-genome comparison (Hagiwara et al., 2014). Accordingly, whole-genome comparison analysis can provide interesting insight into genetic variation provoked during infection, and is therefore a powerful tool for further understanding genome-scale azole resistance mechanisms.

## Frontiers of azole resistance in non-*fumigatus Aspergillus* species

As is the case with *A. fumigatus*, acquired antifungal resistance is potentially able to arise in non-*fumigatus* species, whether in the environment or within a host. However, reports identifying antifungal resistance in such species are so far limited. Genome data for several *Aspergillus* species are available in databases, and the genomes of the cryptic species *A. lentulus* and *A. udagawae* as well as *A. calidoustus* were recently added (Kusuya et al., 2015, 2016; Horn et al., 2016). Thus, there is an opportunity to study pathogenic *Aspergillus* genomes with special reference to azole resistance. The characteristics of pathogenic *Aspergillus* genomes are shown in Table 4.

**Table 4:** **Features of pathogenic ***Aspergillus*** genomes**.

**Species**	**Strain**	**Size (Mb)**	**GC%**	**Protein-coding genes**	**Cyp51 genes**	**Cdr1B genes**	**Reference database**
*A. fumigatus*	Af293	29.42	49.8	9840	2	1	AspGD
*A. flavus*	NRRL 3357	36.89	48.3	13,485	3	2	NCBI
*A. niger*	CBS 513.88	33.98	50.4	14,058	2	2	AspGD
*A. tubingensis*	CBS 134.48	35.15	49.2	12,322	2	2	JGI
*A. terreus*	NIH2624	29.36	52.8	10,401	2	3	NCBI
*A. fischeri*	NRRL 181	31.77	49.5	10,395	2	1	NCBI
*A. lentulus*	IFM 54703	30.96	49.5	9680	2	1	NCBI
*A. udagawae*	IFM 46973	32.19	49.6	9999	2	1	NCBI
*A. calidoustus*	SF006504	41.10	51.1	15,139	2	2	NCBI

**Figure 3:**
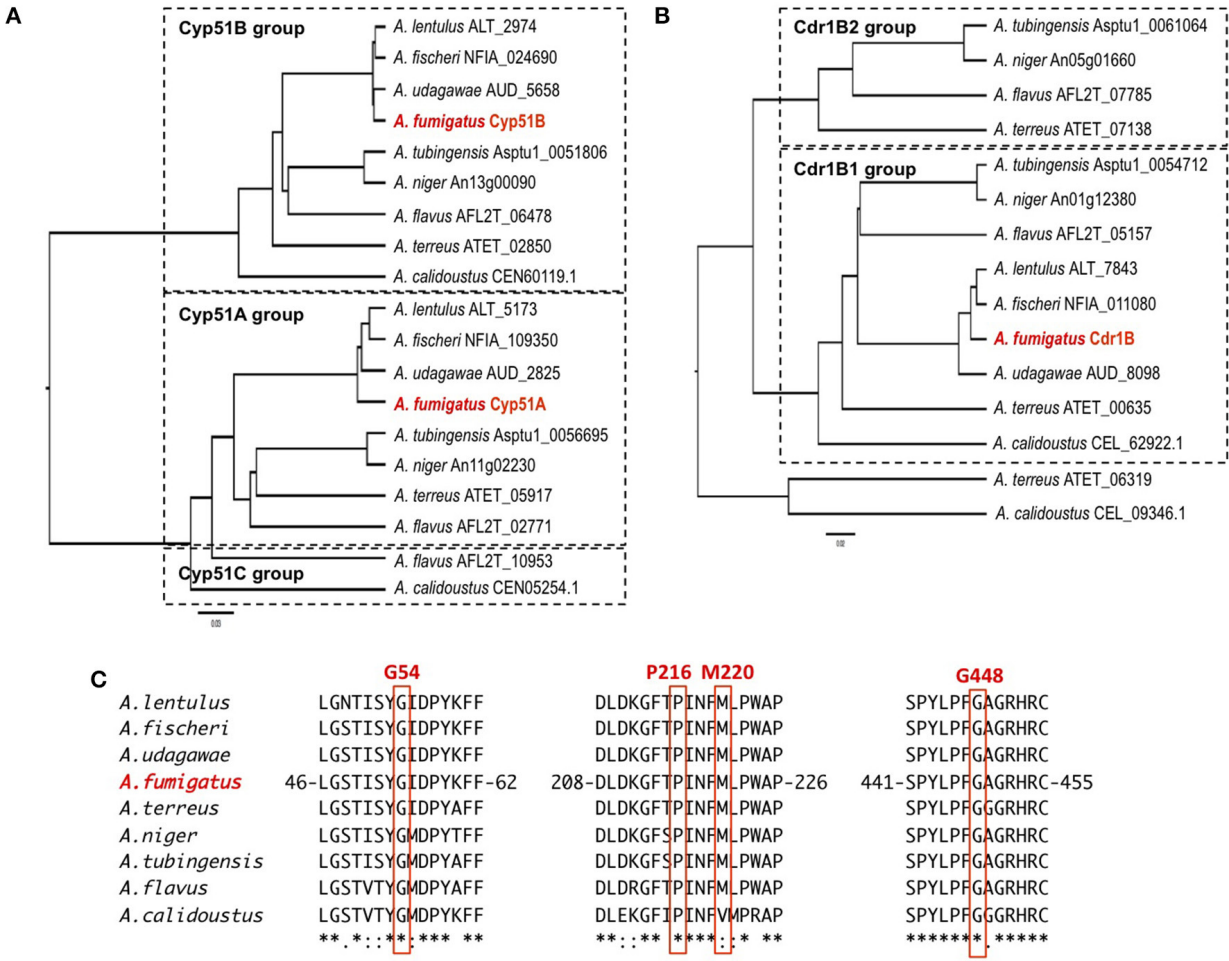
**The molecular genetic phylogenetic trees of the Cyp51A and Cdr1B proteins of pathogenic ***Aspergillus*** fungi**. The sequences were retrieved from the AspGD and NCBI databases according to sequence similarity. The protein sequences of Cyp51A **(A)** and Cdr1B **(B)** were aligned using the ClustalW software, and the phylogenetic trees were constructed by the UPGMA method. The trees were drawn using FigTree v1.4.2 software. The IDs shown behind a species name are associated with the database from which the sequences were retrieved. **(C)** Amino acid sequence alignment of Cyp51A. The sequence surrounding the azole resistance-related amino acids (G54, P216, M220, and G448) is depicted. The numbers indicate amino acid position in the *A. fumigatus* Cyp51A protein.

The sequences of the Cyp51 and Cdr1B proteins of *A. flavus, A. niger, A. tubingensis, A. terreus, A. fischeri, A. lentulus, A. udagawae, A. calidoustus* as well as *A. fumigatus*, were retrieved from the NCBI (http://www.ncbi.nlm.nih.gov/) and AspGD (http://www.aspgd.org/) databases and compared. Phylogenetic trees constructed based on the Cyp51 and Cdr1B protein sequences are depicted in Figures 3A,B, respectively. All of the species listed above, except for *A. flavus*, possess two Cyp51 proteins, Cyp51A and Cyp51B, which form distinct sub-groups in the phylogenetic tree. The third Cyp51 protein, Cyp51C, is found only in *A. flavus*, and is relatively similar to Cyp51A (Figure 3A). The Y319H mutation in *A. flavus* Cyp51C was found specifically in an azole-resistant clinical isolate, suggesting a role in the resistance mechanism (Paul et al., 2015). Through an *in vitro* evolution experiment, several mutations in Cyp51A (K197N, D282E, M288L, Y132N, and T469S) and Cyp51B (H399P, D411N, T454P, and T486P) were identified in multi-azole-resistant *A. flavus*, which warranted further study (Krishnan-Natesan et al., 2008). It is interesting to note that intrinsically azole-resistant species *A. calidoustus* has two Cyp51 proteins. One of these, a Cyp51A-like protein, shows relatively distant homology to *A. fumigatus* Cyp51A, whereas the other falls into the Cyp51B group (Figure 3A). When the *A. calidoustus* Cyp51A-like protein was aligned with Cyp51A proteins from other *Aspergillus* species, the methionine at position 220 in *A. fumigatus* Cyp51A was replaced with a valine in *A. calidoustus* Cyp51A (Figure 3C). As M220 in the *A. fumigatus* Cyp51A protein is involved in azole resistance, this replacement may cause the observed insensitivity to azole drugs in this fungus. It is yet to be confirmed whether the varied azole susceptibility among species is indeed derived from the sequence difference in the Cyp51A protein. Regarding the protein sequences of Cyp51A and Cyp51B in *A. lentulus*, differences of 16 and 12 amino acids, respectively, were detected compared with the corresponding sequences in *A. fumigatus*. Mellado et al. showed that heterologous expression of the *A. lentulus cyp51A* gene in the *A. fumigatus cyp51A* deletion mutant resulted in an *A. lentulus* level azole-resistant phenotype (Mellado et al., 2011). This elegantly demonstrated that Cyp51A is responsible for the differences in azole resistance between *A. fumgiatus* and *A. lentulus*.

As described above, *A. fumigatus* has 12 ABC transporters in a PDR sub-group that includes the Cdr1B protein. Likewise, according to sequence similarity, *A. flavus* and *A. niger* have 13 and 15 PDR-type ABC transporters, respectively (Table 5). The Cdr1B ortholog is duplicated in *A. flavus* and *A. niger*. From the genome sequence data, one Cdr1B protein is present in *A. fumigatus, A. fischeri, A. lentulus*, and *A. udagawae*, whereas *A. flavus, A. niger, A. tubingensis*, and *A. calidoustus* possess two Cdr1B proteins, and *A. terreus* has three such proteins (Figure 3B). The distribution of Cdr1B proteins therefore varies among *Aspergillus* species/complexes.

**Table 5:** **List of the PDR-type ABC transporter genes**.

***A. fumigatus***	**Protein length**	***A. flavus***	**Protein length**	***A. niger***	**Protein length**
**Gene ID**		**Gene ID**		**Gene ID**	
Afu1g14330 (*cdr1B*)	1498	AFL2T_01689	1367	An01g03900	1355
Afu1g17440	1454	AFL2T_03236	1482	An01g08720	1976
Afu2g15130	1500	AFL2T_03320	1408	An01g12380 (*cdr1B1*)	1540
Afu3g01400	1425	AFL2T_03503	1468	An05g01660 (*cdr1B2*)	1496
Afu3g07300 (*atrI*)	1502	AFL2T_05157 (*cdr1B1*)	1495	An06g02550	1336
Afu4g01050	1350	AFL2T_05761	1520	An07g01250	1442
Afu5g00790	1472	AFL2T_07664	1491	An08g03300	1456
Afu5g02260	1470	AFL2T_07785 (*cdr1B2*)	1499	An08g04500	1474
Afu5g09460	1476	AFL2T_07845	1445	An11g02110	1490
Afu6g04360 (*atrF*)	1548	AFL2T_07984	1410	An13g03060	1421
Afu6g08020	1527	AFL2T_09480	1334	An13g03570	1478
Afu8g02650	1454	AFL2T_10593	1420	An14g02610	1358
		AFL2T_11475	1518	An14g03570	1433
				An15g01130	1536
				An15g02930	1491

## Conclusion and perspectives

Recent genetic and genomic studies have provided important insights into azole resistance mechanisms in *Aspergillus*. In particular, whole-genome comparison has proven to be a powerful tool for discovering novel mutations responsible for drug resistance. The analysis of genome sequences has also advanced our understanding of the important diversity of *Aspergilli*. However, there remains much to be investigated in *A. flavus* and *A. niger*, the second most frequent causative agents of aspergillosis. A comprehensive investigation of the genes related to azole resistance among a large population of clinical isolates would broaden our knowledge. In addition, the variable drug susceptibility within the cryptic species may form an important focus of future studies. A more complete understanding of the mechanisms underlying azole resistance will aid the development of new therapeutic drugs against azole-resistant *Aspergillus* fungi/strains.

## Author contributions

DH, AW, KK, GG: Designed the study. DH: Wrote the manuscript.

## Funding

This work was also supported in part by the Japanese Ministry of Education, Culture, Sports, Science, and Technology (MEXT) Special Budget for Research Project: The Project on Controlling Aspergillosis and the Related Emerging Mycoses. GG thanks for Fundação de Amparo a Pesquisa do Estado de São Paulo (FAPESP) and Conselho Nacional de Desenvolvimento Cientifico e Tecnologico (CNPq), both from Brazil, for providing resources for the research in his laboratory.

### Conflict of interest statement

The authors declare that the research was conducted in the absence of any commercial or financial relationships that could be construed as a potential conflict of interest.
